# A Survey of Information Entropy Metrics for Complex Networks

**DOI:** 10.3390/e22121417

**Published:** 2020-12-15

**Authors:** Yamila M. Omar, Peter Plapper

**Affiliations:** Faculty of Science, Communication and Medicine, University of Luxembourg, L-1359 Luxembourg, Luxembourg; peter.plapper@uni.lu

**Keywords:** complex networks, entropy, centrality, Shanon’s entropy

## Abstract

Information entropy metrics have been applied to a wide range of problems that were abstracted as complex networks. This growing body of research is scattered in multiple disciplines, which makes it difficult to identify available metrics and understand the context in which they are applicable. In this work, a narrative literature review of information entropy metrics for complex networks is conducted following the PRISMA guidelines. Existing entropy metrics are classified according to three different criteria: whether the metric provides a property of the graph or a graph component (such as the nodes), the chosen probability distribution, and the types of complex networks to which the metrics are applicable. Consequently, this work identifies the areas in need for further development aiming to guide future research efforts.

## 1. Introduction

A wide range of problems, such as social network analysis, communications routing, protein interactions, identification of key players in transaction networks, vulnerability of water distribution networks, and city traffic studies, deals with determining the entropy of relational structures, such as complex networks or graphs. Given the great differences in routes and flow types that these varied networks follow, a great body of research has been developed that presents varied information entropy metrics. Each entropy metric provides different characteristics of a network or its components. Furthermore, it is accepted that not all of the metrics are applicable to all networks. Unfortunately, this growing body of research is scattered in multiple disciplines. Thus, it is difficult to identify the available metrics and understand the context in which they are applicable, as well as to determine areas in need of further development. Consequently, a systematic literature review of information entropy metrics for graphs is direly needed. This work aims at conducting a survey of existing graph entropy metrics that are specifically based on information entropy, as described by Shannon’s formulation [1]. In addition, these entropy metrics will be classified with respect to the probability distribution that they make use of and the type of networks they can be applied to. The final objective of this work is to identify open research avenues.

This work is organized, as follows. Section 2 introduces preliminary concepts in graph theory and Shannon’s information entropy. This section provides definitions, formulations and notation used throughout this work. Section 3 details the procedure that is followed in order to produce this review article and defines the scientific questions that this work aims to answer. Section 4 summarizes the works that were considered for this narrative review in terms of three factors: the use of entropy as a networks vs. a node metric, the different types of probability distributions, and the types of graphs to which these metrics are applicable. Finally, Section 5 discusses open research avenues and presents final remarks.

## 2. Preliminaries

### 2.1. Complex Networks

A complex network or graph *G* is composed of a finite set of nodes *V* and a set of edges *E*. The set of nodes cannot be empty, V≠∅, thus $V=\{v_{0},v_{1},...,v_{N}\}$. The set of edges *E* are pairs of nodes $\left(v_{i},v_{j}\right)$ that denote some kind of relationship between nodes. Two nodes that are joined by an edge are referred to as adjacent or neighboring. If the edges are unordered, then the graph is said to be undirected. When the edges are ordered, the graph is called directed. Simply put, in an undirected graph, the edges $\left(v_{i},v_{j}\right)$ and $\left(v_{j},v_{i}\right)$ are equivalent, while, in a directed graph, they are not.

Graph *G* can be completely described by means of an adjacency matrix A of elements $a_{ij}=1$ if there exists an edge between nodes $v_{i}$ and $v_{j}$ and 0 otherwise. Such graphs are known as binary or unweighted. If the edges carry a numerical value measuring a property of the edge (distance, strength of a relationship, number of transactions, etc.), then graph *G* is generally described while using a weight matrix W of elements $w_{ij}>0$ and it is called a weighted graph.

A graph *G* is said to be connected, if, for every pair of distinct nodes $v_{i}$ and $v_{j}$, there is a path from $v_{i}$ to $v_{j}$; otherwise, it is said to be disconnected.

### 2.2. Notation

In this work, the node notations $v_{i}$ and *i* are considered to be equivalent, $v_{i}\equiv i$, and it used interchangeably in order to avoid multiple subscripts where necessary.

### 2.3. Traditional Centrality Metrics

#### 2.3.1. Degree

For an undirected graph, the degree of a node *i* is the number of nodes *j* to which *i* is adjacent. The degree $k_{i}$ can be calculated from the adjacency matrix, as follows:

(1) $$k_{i}=\sum_{j}a_{ij}=\sum_{j}a_{ji}.$$

For directed graphs, the in- and out-degree, $k_{i}^{\mathrm{in}}$, and $k_{i}^{\mathrm{out}}$, respectively, can be defined. The former is the number of ingoing links, while the latter indicates the number of outgoing links. In general, the degree can be calculated as the sum of these two components [2]:

(2) $$k_{i}=k_{i}^{\mathrm{in}}+k_{i}^{\mathrm{out}}=\sum_{j}a_{ji}+\sum_{j}a_{ij}.$$

#### 2.3.2. Strength

The node strength is the natural generalization of the node degree for weighted graphs. Thus, it can be calculated from the weight matrix. For undirected graphs, it is defined as

(3) $$s_{i}=\sum_{j}w_{ij}=\sum_{j}w_{ji}$$

and for directed graphs, as follows

(4) $$s_{i}=s_{i}^{\mathrm{in}}+s_{i}^{\mathrm{out}}=\sum_{j}w_{ji}+\sum_{j}w_{ij}.$$

#### 2.3.3. Betweenness

The betweenness centrality η was defined by Freeman [3] and it represents the fraction of times in which a node *v* falls on the geodesic path σ between any two other nodes *i* and *j*. This metric captures the potential that node *v* has to control the communication between nodes *i* and *j*. It can be calculated as

(5) $$\eta_{v}=\sum_{i,j\in V}\frac{\sigma (i,j|v)}{\sigma (i,j)}.$$

Note that a geodesic path is the shortest possible sequence of linked nodes from *i* to *j*, in which neither nodes nor edges are repeated.

#### 2.3.4. Closeness

Closeness centrality is defined in terms of distance and it can be interpreted either as a metric of independence from control by others or as a measure of access or efficiency [4]. Given d(i,j), i.e., the distance between nodes *i* and *j*, ∀j∈V, the closeness centrality *C* of node *i* is defined as

(6) $$C_{i}=\frac{1}{\sum_{j\in V}d\left(i,j\right)}$$

#### 2.3.5. Eigenvector

Being originally suggested by Bonacich [5,6], eigenvector centrality uses the eigenvector of the largest eigenvalue of the adjacency matrix A as a centrality metric. While degree centrality weights every contact equally, the eigenvector weights neighbors according to their own value of centrality, i.e., the centrality of node *i* is proportional to the sum of the centralities of the nodes to which it is connected. This metric can also be interpreted as a weighted sum of not only direct neighbors, but indirect ones of every length. The eigenvector centrality is defined as

(7) $$x_{i}=\frac{1}{\lambda }\sum_{j=1}^{N}a_{ij}x_{j}$$

where λ is the largest eigenvalue of $A=a_{ij}$ and *x* is the corresponding eigenvector.

#### 2.3.6. Clustering Coefficient

The clustering coefficient, which was first introduced in [7], indicates that the likelihood that two neighbors of a node *i* are adjacent, i.e., the ratio between the number of triangles $t_{i}$, with *i* as one vertex and the number of all possible triangles that *i* could form $T_{i}$.

(8) $$CC\left(i\right)=\frac{t_{i}}{T_{i}}=\frac{2t_{i}}{k_{i}\left(k_{i}-1\right)}.$$

The original formulation [7] is applicable in the case of binary undirected networks. Several generalizations were made in order to extend its application to weighted undirected networks [8] as well as to both binary and weighted directed networks [9].

### 2.4. Information Functional

Dehmer [10] defined an information functional *f* of graph *G* as a function that is capable of capturing structural information of the underlying graph. $f:S\to R_{+}$ where *S* is an abstract set. In addition, *f* is assumed to always be monotonous. Because *f* has to be defined concretely, arbitrary graph-theoretical properties or quantities can be used. Thus, an information functional *f* can be, for example, any of the centrality measures defined in the previous Section.

An information functional quantifies structural information of a graph and produces a derived probability distribution, as follows

(9) $$p\left(v_{i}\right)=\frac{f(v_{i})}{\sum_{j=1}^{N}f\left(v_{j}\right)}$$

where *f* is an arbitrary information functional. Because $0\leq p(v_{i})\leq 1$ and $\sum_{i=1}^{N}p\left(v_{i}\right)=1$, the quantities $p(v_{i})$ can be interpreted as vertex probabilities.

### 2.5. Shannon’s Entropy

Information theory originated in the need to quantify fundamental limits on signal processing. Shannon [1] developed the concept of information entropy, which quantifies the average number of bits needed to store or communicate a message: one cannot store or communicate a message with *n* different symbols in less than $\log_{2}n$ bits. Shannon’s entropy determines a lower limit, below which no message can be further compressed. In addition, Shannon’s information theory has also been regarded as a measure to quantify uncertainty, or entropy, in a system [11]. It allows for quantifying the uncertainty that is involved in predicting the value of a random variable, i.e., the amount of randomness or freedom of choice. It is defined, as follows:

**Definition** **1.** *For an ensemble $X(R,p_{i})$, where R is the set of possible outcomes (the random variable), and n=|R| and $p_{i}$ is the probability of an outcome in R. The Shannon information content or entropy of X is given by*

(10) $$H\left(X\right)=-\sum_{i=1}^{n}p_{i}\log_{2}p_{i}$$

*where calculating H(X) requires the mass distribution probability of ensemble X.*

However, it must be noted that the Shannon’s information measure can be based on logarithms of base 2, *e* or 10 without loss of generality.

Shannon’s information entropy formulation has a number of properties worth mentioning:

- Because Shannon’s information entropy is a measure of uncertainty, the entropy *H* increases as the probabilities $p_{i}$ become equal. In fact, *H* attains its maximum possible value when all of the $p_{i}$ are exactly equal. In this case, where all $p_{i}=1/n$, $H=\log_{2}n$.
- For equiprobable outcomes, the value of the entropy *H* increases with *n*.
- When there is only one possible outcome, the system is perfectly predictable and, thus, H=0.
- The mathematical formulation that is presented in Equation (10) is that of a continuous function of $p_{i}$. Additionally, thus, small changes in $p_{i}$ result in small changes in *H*.
- Equation (10) is a symmetric function, i.e., exchanging the values of two probabilities does not change the resulting value of the entropy *H*.

### 2.6. Other Definitions

#### 2.6.1. Paths, Geodesics, Walks and Trails

Borgatti [12] explained that flow on a network can follow different types of routes. He classified them, as follows. *Paths* are a sequence of linked nodes in which neither nodes nor edges are repeated. A path of length *n* from node *i* to node *j* is an ordered sequence of distinct nodes $P=\{v_{0},v_{1},...,v_{n}\}$ with $v_{0}=i$, $v_{n}=j$ and $(v_{t},v_{t+1})\in E$ for t=0,1,...,n−1. *Geodesics* are shortest paths. The notation is analogous to paths, with the caveat that *P* is the shortest path from *i* to *j*. *Trails* allow for nodes to be repeated; however, each edge $\left(v_{t},v_{t+1}\right)$ can appear only once. Finally, *walks* allow for both nodes and edges to be repeated.

#### 2.6.2. Distance in Graph *G*

The distance between two nodes *i* and *j* in graph *G* is written as d(i,j) and it corresponds to the number of edges in a shortest path, i.e., the geodesic, connecting them.

#### 2.6.3. Graph Diameter

The diameter of a graph *G* is the greatest distance between any two vertices in *G*. It is denoted as $D\left(G\right)=\max_{i\in V}\max_{j\in V}d\left(i,j\right)$.

## 3. Materials and Methods

**Figure 1:**
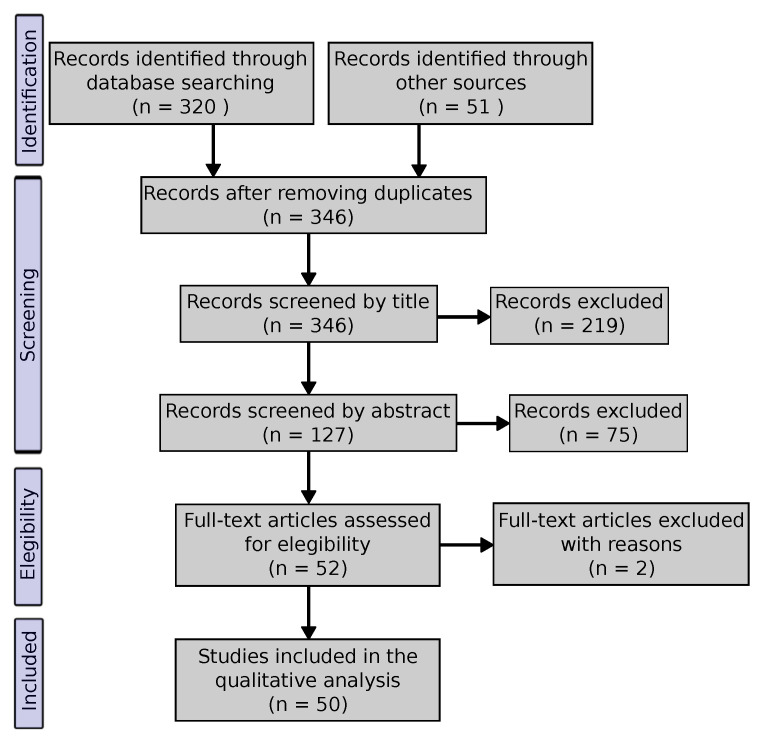
Preferred Reporting Items for Systematic Reviews and Meta-Analyses (PRISMA) flow diagram describing the systematic review process undergone in this work.

The present literature synthesis is the result of a systematic review that was conducted following the Preferred Reporting Items for Systematic Reviews and Meta-Analyses (PRISMA) flow diagram [13] in Figure 1. The original database search was conducted in July 2020 while using Web of Science. The search terms were “entropy AND centrality” conducted on title, abstract, author keywords, and Keywords Plus. All of the years and indexes were used. Only records that were in English language were eligible.

The “other sources” are mostly constituted by articles identified through Google Scholar. Any other articles referenced in those identified through database search and deemed interesting were further explored and they are also accounted in the “other sources” set.

After removing duplicate records, these were screened by title and then by abstract to identify records dealing with entropy in the context of complex network analysis. Full text assessment was conducted on 52 records and 50 of them were included in the study that was presented in this work. These two records were excluded, because they use Shannon’s entropy in order to calculate the weight coefficients of a decision matrix instead of using it to calculate the entropy of the nodes or network itself.

The present study focuses on identifying a number of elements in the records evaluated:

- Is Shannon’s entropy used as a network or node metric?
- When Shannon’s entropy is used with the objective of ranking nodes by importance (also known as a centrality metric), is the entropy of the node calculated directly or obtained as the difference between the entropy of the graph before and after node removal?
- What is the definition of the probability distribution $p_{i}$?
- To which type of complex network is the metric applicable? Undirected or directed graph? Weighted or unweighted? Should the graph be strongly connected? Are self-loops allowed? And so on.

## 4. Results

**Figure 2:**
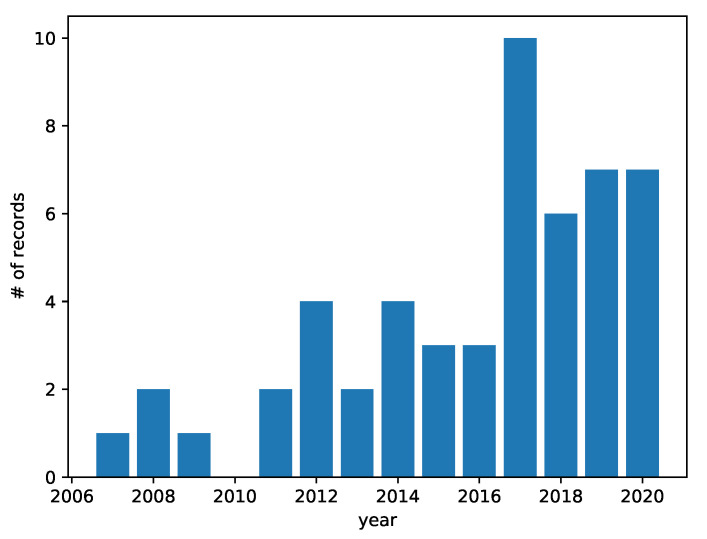
Number of articles included by year.

**Figure 3:**
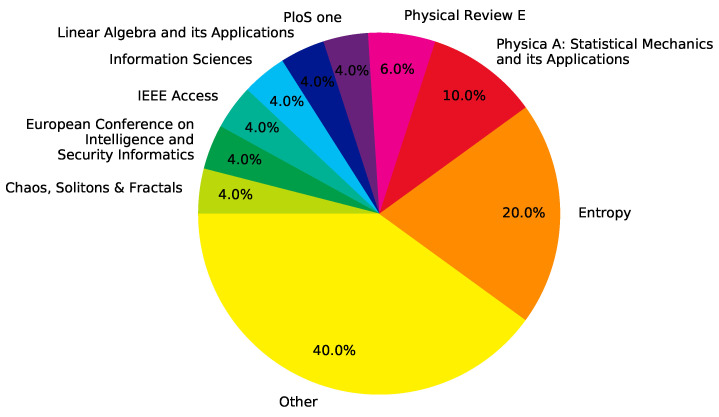
Distribution of records by journal.

The 50 records that are included in this literature review are summarized, in chronological order, in Table 1. The 50 included articles span close to 15 years, from 2007 to date. While research on the use of entropy as a centrality metric for complex network analysis is on the rise, 2017 has been the most prolific year to date, as shown in Figure 2. Furthermore, the included articles were authored by over 130 people. No particular author stands out, since the most prolific researchers in this subject authored, at most, three articles on the topic. Journal articles represent 80% of included records, while conference proceedings account for 20%. Finally, Figure 3 shows the distribution of the articles on different journals. In the following Sections, answers to the research questions that are introduced in Section 3 are presented.

### 4.1. Shannon’s Entropy as a Networks vs. Node Metric

#### 4.1.1. Graph Entropy

A number of authors use information entropy as a network or graph metric [15,16,18,22,23,24,25,26,27,28,31,34,35,37,38,39,40,41,50,53]. However, a distinction should be made: while all of these works measure the entropy of the network, some use graph entropy to indirectly measure the centrality of the nodes (see Section 4.2). In the latter, the total entropy of the full network, as well as the entropy of the network when node *i* and its edges had been removed are calculated. Subsequently, the entropy change ΔH(i)=H(G)−H(G−{i}) produced by node *i* can be obtained ∀i∈V. In these works, the authors claim that the maximum graph entropy change is associated with the most central node.

Traditional graph entropy metrics are based on graph invariants, such as the number of nodes or edges or the degree distribution [18]. These metrics are typically used to measure the structural complexity of a graph. A limitation of these graph entropy metrics is that structurally non-equivalent graphs may have the same information content, i.e., the same value of graph entropy.

It is also possible to produce graph entropy metrics that are based on information functionals [18,22] (see the definition in Section 2.4). In fact, probability distribution definitions while using information functionals are used in several graph entropy metrics. For example, information functionals are based on edge or node betweenness centrality [24,25,34,50,53] distances to a given vertex [28], degree, degree power or probability distribution of degrees [31,41], paths or paths’ length [16,35], and closeness or eigenvector centrality [53].

#### 4.1.2. Node Entropy

Information entropy may also be used as a centrality metric to rank nodes (or edges) by importance. In this sense, several authors [14,17,19,20,21,23,29,30,32,33,36,42,43,44,45,46,47,48,49,50,51,52,54,55,56,57,58,59,60,61,62,63] developed a number of node entropy metrics and provided the appropriate interpretation. Like for graph entropy metrics, many of these are constructed equivalently to the use of information functionals (see Equation (9)) [20,21,44,48,51,56,59,60,61,62]. Node entropy metrics are based on degree [21,32,56,57,59,60,62,63], neighbor degree [59] or strength [57], the weight of edges [56,60], betweenness [21,33], closeness centrality [21], paths [14,17,54], or walks [19,23,29,30], or less traditional metrics, such as the topological potential [20], the probability of information flow [36], the probability of a protein complex [55], the number of nodes [58], or protein annotations [61]. In many cases, locality is important and, thus, the properties of the neighbors are taken into account [57,60,62,63].

### 4.2. Centrality of Nodes: Direct vs. Indirect Entropy Metrics

As explained in the previous Section, entropy metrics can be used as centrality metric in a similar fashion to degree or betweenness centrality. This type of entropy metrics produces node rankings and aids in the identification of the most central node (according to a context-dependent definition of centrality). These rankings can be of two types: direct and indirect. The former implies the calculation of node entropy H(i) directly from a probability distribution. The latter is obtained from the change in entropy that is incurred when a node and its adjacent edges are removed from a graph. In this case, the importance of a node is calculated as the difference between the baseline (or full) graph entropy and that of the graph once node *i* and its edges have been removed, H(i)=H(G)−H(G−{i}). Section 4.1.1 previously noted this, where it was stated that the entropy of the network H(G) is sometimes used to indirectly measure the centrality of nodes. Section 4.1.2 lists examples of direct node entropy centrality metrics, while examples of indirect node entropy rankings are available in [15,16,21,39].

It should be noted that, like traditional centrality metrics, node rankings that are based on entropy are calculated based on a number of assumptions as well as the characteristics of the underlying graph. Consequently, not all centrality metrics are applicable to all graphs. This was originally stated by Borgatti [12], who explained that flow on a network must follow a type of route (paths, geodesics, trails, or walks) and a type of traffic (parallel duplication, serial duplication, or transfer). Indeed, several authors echoed the fact that the meaning of centrality is context-dependent in the cited works:

- “Each measure of centrality makes assumptions about the importance of the various types of traffic flow and, thus, each measure of centrality can be assessed by where it falls in the typology (refers to Borgatti’s typology [12])” [14]
- “Key players are those elements in the network that are considered to be important, in regard to some criteria.” [16]
- “... in any centrality application one should take into account the characteristics of the flow of traffic through the network.” [17]
- “Choosing the right centrality for a specific problem is usually a hard task and [a] common approach is comparing different centralities for the same network and building hypothesis about the discovered central nodes.” [21]
- “Different measures of centrality capture different aspects of what it means for a node to be ‘central’ to the network.” [29]
- “A centrality is optimal for one application, yet is often sub-optimal for a different application.” [32]
- “Centrality is an important concept in network theory, yet there is no unique definition.” [37]
- “Centrality is a measure of the importance of a node in a complex network with respect to a specific criterion, where several centrality criteria have been proposed.” [40]
- “... a specific centrality is ideal for one application, yet regularly imperfect for an alternate application.” [44]
- “The significance [the authors mean importance] of a node can have different meanings depending on its application.” [45]
- “What most important’ means is not universally defined, therefore numerous notions of centrality have been proposed.” [46]
- “[centrality measures] ... are intended to capture the role played by each node within the network by optimizing an opportunely defined objective function.” [49]
- “The meaning of ‘important’ depends on the nature of the problem analyzed.” [54]
- “... all of these methods [centrality metrics] have some limitations and specific application scenarios that are related to the way they consider the problem. A valid method for ranking nodes in a complex network remains an open issue.” [57]
- “In general, each network has a specific node importance ranking, and different identification methods consider different structural properties of the network, which would give different ranking lists.” [58]
- “Various centrality metrics establish different aspects for the meaning of an actor to be central to the network.” [63]

### 4.3. Probability Distributions

The probability distribution $p_{i}$ can be based on different graph attributes. The most commonly encountered ones are:

1. degree or strength (18 records),
2. betweenness (eight records),
3. paths (five records),
4. walks (six records),
5. closeness (four records),
6. distance (three records),
7. eigenvector (two records), and
8. other definitions (nine records).

#### 4.3.1. $p_{i}$ Based on Node Degree

By far, the largest number of entropy metrics are based on node degree [16,21,31,39,41,42,49,56,62], its extension to weighted graphs, i.e., node strength [38,56], the degree and/or strength of neighbors of a node [32,43,57,59,60,63], or degrees associated to a subgraph of a node [44,48]. Yet, the definition of the probability distribution varies from one author to another. A brief summary of these metrics is provided hereafter, and their formulation is presented in a number of tables.

#### $p_{i}$ Based on the Degree of Node *i*

The first group corresponds to entropy metrics, where the probability distribution $p_{i}$ is based on the degree of the node *i*, i.e., $k_{i}$. Table 2 summarizes these.

Ortiz-Arroyo et al. [16] defined $p_{i}=k_{i}/\sum_{j}k_{j}=k_{i}/2N$, aiming to determine the connectivity of a node in a graph by calculating the baseline entropy as well as the entropy of the graph, where node *i* has been removed. In Serin et al.’s work [21], $p_{i}=k_{i}^{\mathrm{norm}}/\sum_{j=1}^{N}k_{j}^{\mathrm{norm}}$ is used, where $k_{i}^{\mathrm{norm}}=\left[k_{i}-k_{\min}\right]/\left[k_{\max}-k_{\min}\right]$ is the normalized degree. Similarly to [16], Serin’s metric is used in order to determine which node removal disconnects the network the most.

Lu et al. [31] used the *q* degree power of node *i*, and defined $p_{i}=k_{i}^{q}/\sum_{j=1}^{N}k_{j}^{q}$. They concluded that, for q=1, the degree entropy of a graph corresponds to the scale of the graph, since this metric increases as the graph grows in the number of nodes and edges. It must also be noted that, when q=1, Lu’s formulation reduces to the degree information functional (see Equation (9)). While Lu et al. [31] does not directly provide a definition of the *q* degree power, they do define the sum of degree powers, as follows $\sum_{q}\left(G\right):=\sum_{i\in V}k_{i}^{q}$, where *q* is an arbitrary real number. In literature [64] it is explained that when q=0, $\sum_{0}\left(G\right)=\sum_{i\in V}k_{i}^{0}=\left|V\right|$ and when q=1, $\sum_{1}\left(G\right)=\sum_{i\in V}k_{i}=2\left|E\right|$.

Likewise, Ai [39] used the information functionals (see Equation (9)) based on the in-, out-, and all-degree. He measured node entropy as the difference between the baseline graph entropy and that of the graph when node *i* is removed. Similarly, Wang et al. [56] used information functionals in order to calculate the structural entropy that is based on the degree, where $p_{i}=k_{i}/\sum_{j}k_{j}$, resembling the work in [31] for q=1.

Cai et al. [41] studied several degree based entropy metrics. The first is the so-called Degree Distribution Entropy (DDE), where $p_{i}=p\left(k\right)$, i.e., the probability distribution is the distribution function of the degree *k*. They also studied the Wu Structure Entropy (WSE), where the definition is equivalent to that of [31] for q=1. Cai et al. [41] also considered two other entropy indices: SD Structure Entropy, where nodes and edges differences determine network heterogeneity, and FB Structure Entropy, where network heterogeneity is determined while using the angle of walk position and medial and radial measurements.

For Wiedermann et al. [42], the entropy of a node is based on the probability to jump between nodes when traveling randomly through a network. The authors used both the adjacency matrix and node degree for this purpose, as follows: $p_{i}=a_{ij}/k_{i}$. Similarly, Tulu et al. [45] defined two probability distributions that were based on the adjacency matrix and the node degree when considering the community to which nodes *i* and *j* belong to. The $p_{i}$ based on the internal density, i.e., $p_{i}=\rho_{i}^{\mathrm{in}}=\sum_{j}a_{ij}/k_{i}$, has both nodes *i* and *j* from community *h*. The external density probability distribution, $p_{i}=\rho_{i,h_{1}}^{\mathrm{ext}}=\sum_{j}a_{ij}/k_{i}$, requires, instead, that both nodes belong to different communities, thus i∈h and $j\in h_{1}$.

Finally, Barucca et al. [49] proposed a metric, called InfoRank, based on the degree sequence allowing for determining the benchmark graph information as well as the node specific information.

#### $p_{i}$ Based on the Strength of Node *i*

A second group of metrics is formed by entropy definitions that are based on node strength $s_{i}$, which, it can be argued, is the extension of node degree to weighted networks. Table 3 summarizes these metrics. Wang et al. [38] calculated the risk of IT projects based on entropy where $p_{i}=s_{i}/\sum_{j}s_{j}$. This risk entropy, they claimed, provides a description of the risk related to heterogeneity of the IT projects. In [56], the authors defined the interaction frequency entropy, which is based on the weight of edges as well as the strength of nodes as follows $p_{i}=w_{ij}/s_{i}$. The latter is the weighted graph equivalent to the work of Wiedermann et al. [42] previously cited. Note that, while the authors in [56] wrote the summation in Shannon’s entropy, as conducted among the neighbors of *i*, $\sum_{j\in \Gamma (i)}$, the result is equivalent to doing $\sum_{j\in V}$, since, for any j∈V, where j∉Γ(i) it is always the case that $w_{ij}=0$. Thus, this is not a truly neighbor strength based entropy metric. Similarly, Ni et al. [60] defined a weight influence entropy while using the same formulation for the probability distribution, as [56].

#### $p_{i}$ Based on the Degree and/or Strength of the Neighbors of Node *i*

Yet another group of entropy metrics can be associated to the degree and/or strength of the neighbors of node *i* (see Table 4). Nie et al. [32] proposed a metric that combines the degree information entropy, defined as $H\left(i\right)=-\sum_{i=1}^{N}k_{i}\log k_{i}$, and the local entropy, $H_{L}\left(i\right)=-\sum_{j\in \Gamma (i)}k_{j}\log k_{j}$, giving rise to the “mapping entropy”. The formulation of the latter is $H\left(i\right)=-k_{i}\sum_{j\in \Gamma (i)}\log k_{j}$. However, we argue that this is not strictly based on Shannon’s entropy. Instead, this metric produces a weighted degree value for node *i* while using the degree of its neighbors and, unlike what is prescribed in Shannon’s formulation (see Equation (10)), the probability $p_{i}$ used before and after the logarithm is not the same as for the other metrics that are presented here.

Zareie et al. [43] proposed an entropy centrality metric that is based on the degrees of the first and second order neighbors. The entropy of the degree of the first order neighbors has $p_{i}=k_{j}/k_{i}^{1}=k_{j}/\sum_{l\in \Gamma (i)}k_{l}$, while the degree entropy of the second order neighbors has $p_{i}=k_{j}^{1}/k_{i}^{2}=\sum_{j\in \Gamma (i)}k_{j}/\sum_{j\in \Gamma (i)}k_{j}^{1}$, respectively. Similarly to Zareie’s entropy of first degree neighbors [43], Guo et al. [59] defined an algorithm that is based on the degree of a node and that of its direct neighbors, where $p_{i}=k_{i}/\sum_{j\in \Gamma (i)}k_{j}$. The main difference is that this algorithm is applied iteratively in order to select influential nodes.

Li et al. [57] proposed a structural entropy centrality made of two components: one that is associated with the in-degree of a node and that of its neighbors, where $p_{i}=k_{i}^{\mathrm{in}}/\sum_{j\in \Gamma (i)}k_{j}^{\mathrm{in}}$; and, another component that is associated with the out-degree where $p_{i}=k_{i}^{\mathrm{out}}/\sum_{j\in \Gamma (i)}k_{j}^{\mathrm{out}}$. Equivalently, Li et al. [57] also defined the interaction entropy that is based on strength with “in” ($p_{i}=s_{i}^{\mathrm{in}}/\sum_{j\in \Gamma (i)}s_{j}^{\mathrm{in}}$) and “out” ($p_{i}=s_{i}^{\mathrm{out}}/\sum_{j\in \Gamma (i)}s_{j}^{\mathrm{out}}$) components.

Ni et al. [60] defined the direct influence as the sum of a weight influence entropy (presented previously in Table 3) and the confidence influence entropy. The latter is calculated while using the degree of node *i*’s neighbors. The probability distribution is defined as $p_{i}=k_{i}^{\beta }/\sum_{j\in \Gamma (i)}k_{j}^{\beta }$, where β is a tunable parameter, called confidence strength.

Wang et al. [62] proposed a probability distribution that is dependent on the degree of the neighbors of node *i*, i.e., $p_{i}=k_{j}/\sum_{l=1}^{N}k_{l}$. This should not be confused with the degree information functional. The authors stated that the summation in Shannon’s entropy formula of Equation (10) is conducted over the neighbors of node *i*, giving $H\left(i\right)=-\sum_{j\in \Gamma (i)}p_{i}\ln p_{i}$.

Finally, Saxena et al. [63] based the entropy metric on the node’s degree as well as the degree of its neighbors, as follows: $p_{i}=1/\left[k_{i}\left(k_{j}-1\right)\right]$. For this probability distribution, $k_{j}$ is the degree of node *j*, which is a neighbor of node *i*, thus j∈Γ(i).

#### $p_{i}$ Based on the Degree and/or Strength of Nodes in a Subgraph of Node *i*

Lastly, and arguably related to the latter group, entropy metrics can be based on degree and/or strength values that are associated with subgraphs of node *i* (see Table 5). Only Qiao et al. [44,48] developed metrics of this kind. Originally developed for undirected, unweighted graphs [44], the local influence can be calculated while using $p_{i}=k_{i}^{G_{i}}/\sum_{j\in G_{i}}k_{j}^{G_{i}}$ where $G_{i}$ is the subgraph in which node *i* is the central node. The metric was later generalized to directed, weighted networks [48]. In this case, the structural information entropy is calculated equivalently to the undirected, unweighted case. The interaction frequency entropy is based on edge weights and out-strength in the subgraph $G_{i}$, as follows $p_{i}=w_{ij}^{G_{i}}/\sum_{l\in G_{i}}w_{il}^{G_{i}}$.

#### 4.3.2. $p_{i}$ Based on Betweenness

A number of authors have based their entropy metric on betweenness centrality. Table 6 presents a summary. Serin et al. [21] proposed a combined metric that is based on degree, betweenness, and closeness (see Section 4.3.1 and Section 4.3.5). The betweenness portion requires $p_{i}=\eta_{i}^{\mathrm{norm}}/\sum_{j=1}^{N}\eta_{j}^{\mathrm{norm}}$, where $\eta_{i}^{\mathrm{norm}}=\left[\eta_{i}-\eta_{\min}\right]/\left[\eta_{\max}-\eta_{\min}\right]$ is the normalized betweenness of node *i*. The authors claim that betweenness entropy can identify nodes that affect the flow of data through the network.

Chellappan et al. [24,25,34] used edge betweenness centrality instead of the node betweennnes. They defined $p_{i}=\eta_{•,•}\left(u,v\right)/\sum_{(x,y)\in E}\eta_{•,•}\left(x,y\right)$, where the double-bullet notation indicates all pairs of nodes. The authors posed that high edge betweenness entropy is indicative of a high diversity of paths in tactical communication networks [24]. They further proposed the use of entropy maximization and betweenness entropy in order to make communications routing decentralized [25] and handle single edge failures [34]. Similarly, Zhang et al. [50] used edge betweenness centrality in order to analyze transportation networks in Xiamen. In these graphs, intersections are represented as nodes, road segments as edges and average travel times as edge weights. The authors proposed $p_{i}=\eta_{i,j}\left(e\right)/\eta_{i,j}$, where e∈E is an edge.

In Ai’s work [39], the importance of a node is measured as the network entropy change before and after node removal. The author used both degree and betweenness (see Section 4.3.1). In both cases, $p_{i}$ is obtained while using information functionals (see Equation (9)), thus $p_{i}=\eta_{i}/\sum_{j=1}^{N}\eta_{j}$. Similarly, Zarghami et al. [53] developed a vulnerability index in order to evaluate water distribution networks. The index is based on betweenness as well as closeness and eigenvector centrality (see Section 4.3.5 and Section 4.3.7). The betweenness portion makes used of information functionals, as in the case of [39].

Gialampoukidis et al. [33] furthered the work of Nie et al. [32] that is described in Section 4.3.1. They developed a metric called Mapping Entropy Betweenness where $H=-\eta_{i}\sum_{j\in \Gamma (i)}\log\eta_{j}$ that they interpreted as a weighted betweenness centrality making use of node *i*’s neighbors. While the authors named this metric “entropy”, just like in the case of Nie et al. [32], this is not strictly based on the formulation of Shannon’s entropy described in Equation (10).

#### 4.3.3. $p_{i}$ Based on Paths

Some authors based their entropy metrics on paths, i.e., a sequence of linked nodes, in which neither nodes nor edges are repeated. Table 7 summarizes these metrics. Tutzauer [14] developed an entropy centrality metric specific for networks that are characterized by a flow that follows paths and corresponds to a transfer process. In this case, $p_{i}=p_{ij}$ is the probability that a flow starting on node *i* ends in node *j*. $p_{ij}$ is given by the sum over all paths between nodes *i* and *j* of the stopping probability in node *j* multiplied by the product of the transfer probability of all the nodes appearing in the path before *j*. The formulation of Tutzauer’s work [14] is available in Table 7. The author furthered his work [17] to define centralization and differentiate it from centrality. He argued that, while centrality is a property of the nodes, centralization is a property of the network. Thus, highly centralized networks have one or few high centrality nodes. In his latter work, Tutzauer [17] continued to use the probability distribution $p_{i}=p_{ij}$ described earlier.

Ortiz-Arroyo et al. [16] proposed, in addition to a degree-based entropy (see Section 4.3.1), a centrality entropy with a probability distribution that is based on the number of paths. In this case, $p_{i}$ is the ratio between the number of paths that have node *i* as a starting point and the total number of paths in graph *G*. The authors calculate the centrality entropy for the full graph and after the removal of a node. They associate the largest change in entropy to the most central node.

Oggier et al. [46] extended Tutzauer’s work [14] to non-atomic flows, i.e., to the case in which the flow can be split among neighbors of *i*, as opposed to fully transferred to a single neighbor. In this case, $p_{i}$ is the sum over all paths between *i* and *j* of the product among all nodes in the path of the split-and-transfer probability times the ratio between the incoming flow and the number of edges it can split to. The authors claim that flow originating at highly central nodes spreads more evenly across the graph. The authors furthered their work in [54], where they defined the split-and-transfer entropy and demonstrated specific applications.

Computational complexity is an important issue that was discussed by several of the authors that developed path-based metrics [14,17,46,54]. This complexity arises from the need to identify all paths in graph *G*. However, the number of paths grows combinatorially as the number of nodes and edges grows. While some solutions that disregard paths whose probability falls under an arbitrary threshold have been reported, these metrics have not had wide adoption for medium to big graphs. Some authors [29] contend that using walks instead of paths is the most computationally efficient alternative, as it is further explained in Section 4.3.4.

#### 4.3.4. $p_{i}$ Based on Walks

An alternative to paths that forgoes the computational complexity are walks. A walk is a sequence of linked nodes, where both nodes and edges can be repeated. A number of authors have used walks to define their entropy metrics. Table 8 summarizes their formulations.

Delvenne and Libert [19] proposed an entropy rank based on Shannon’s entropy for Ruelle–Bowens random walks of length *t*. The authors determined that the probability $p_{i}=p_{ij}$ of a random surfer following a walk of length *t* from node *i* to node *j* does not depend on the intermediate vertices. In fact, $p_{ij}=\lambda^{-t}u_{i}v_{j}$, where λ is the dominant eigenvalue of the adjacency matrix of maximal magnitude, *u* is the non-negative left eigenvector for λ, and *v* is the non-negative right eigenvector for λ. They claim that Ruelle–Bowens random walks provide a spectral centrality that is different from those that are found in literature and which properties may be more suitable in certain contexts.

Fewell et al. [23] used a number of network metrics in order to analyze the 2010 NBA first round play-offs. In particular, they calculated individual player entropy (while using Shannon’s entropy formulae) in order to measure the uncertainty of ball transitions between any player or outcome. They also estimated “team entropy”, which measures the multiplicity of options across all ball movements instead of just across players, from the transition matrix describing ball movement probabilities. The authors proposed that entropy is strongly influenced by the extent to which multiple players distribute the ball. In fact, their study demonstrated that high team entropy was a good predictor of team success. However, their work does not provide sufficient information to enable the reproducibility of the metric.

Estrada et al. [26] defined a walk entropy that measures the uncertainty in selecting a walk starting at node *i* and finishing at the same node. It is understood as a measure of how much a walker is localized in a few nodes. The probability distribution represents the probability of selecting at random a closed walk among all such walks in the graph, thus $p_{i}={\left[\exp\left(k_{B}^{-1}T^{-1}A\right)\right]}_{ii}/Z$, where A is the adjacency matrix, ${\left(k_{B}T\right)}^{-1}$ is the inverse temperature and $Z=\mathrm{Tr}[\exp\left(k_{B}^{-1}T^{-1}A\right)]$ is the partition function for the graph. Benzi [27] furthered Estrada et al.’s works [26] by providing mathematical proof to a conjecture that was formulated by the latter regarding walk entropy in walk-regular graphs.

Nikolaev et al. [29] furthered Tutzauer’s work [14] by relaxing the requirement to find all paths in graph *G* and using walks of length *t* instead. This method solves computational efficiency issues, given that the entropy of node *i* for a walk of length *t* can be calculated exactly while using matrix multiplication. For this, the authors defined $p_{i}=p_{ij}^{\left(t\right)}+p_{ij^{\prime }}^{\left(t\right)}$, where $p_{ij}$ are the elements of the Markov chain transition probability matrix. Yet, it must be noted that following Borgatti’s classification of flow [12], Nikolaev et al.’s, and Tutzauer’s work are not applicable to the same typology of flow.

Caravelli’s work [30] also introduced a walk based entropy metric for nodes. The metric aims to determine the walk complexity that is attached to a node, given by all possible walks that originate from it. It uses Markov trajectories in order to calculate the entropy of node *i*, where $p_{i}=M_{ij}^{*}=\lim_{k\to \infty }\sum_{j_{1},...,j_{k}=1}^{N}M_{ij_{1}}...M_{j_{k}j}$, where $M_{ij}$ is the Markov operator.

#### 4.3.5. $p_{i}$ Based on Closeness

As discussed previously, Serin et al. [21] proposed a combined metric that was based on degree, betweenness, and closeness (see Section 4.3.1 and Section 4.3.2). The closeness portion requires $p_{i}=C_{i}^{\mathrm{norm}}/\sum_{j=1}^{N}C_{j}^{\mathrm{norm}}$, where $C_{i}^{\mathrm{norm}}=\left[C_{i}-C_{\min}\right]/\left[C_{\max}-C_{\min}\right]$ is the normalized closeness (see Table 9). The authors claimed that closeness entropy can pinpoint the nodes that have the greatest effect on global connectivity when removed.

Dehmer et al. [22] studied the relations between existing entropy measures that are based on information functionals (see Equation (9)). Thus, $p_{i}=C_{i}/\sum_{j}C_{j}$ (see Table 9). Their work is applicable to simple undirected graphs. The authors focused on special classes of graphs, such as stars, paths graphs and union and join of graphs. Similarly, Zarghami et al. [53] developed a vulnerability index to evaluate water distribution networks. The index is based on betweenness as well as closeness and eigenvector centrality (see Section 4.3.2 and Section 4.3.7). The closeness portion makes use of information functionals (see Equation (9)), where $p_{i}=C_{i}/\sum_{j}C_{j}$, like in the case of [22] (see Table 9).

Finally, Wang et al. [38] developed a combined metric. The first part is calculated while using the probability distribution that was obtained from the sum of the clustering coefficient $C_{C}$ and the closeness centrality $C_{i}$ values. Thus, $p_{i}=p_{k}=\sum_{j}p\left(\mu_{j}\right)$, where $\mu_{j}\in \Omega_{k}$ and $\mu_{j}=\beta_{1}C_{j}+\beta_{2}C_{C}\left(j\right)$. The range of μ is [0,1] and it is divided into ten intervals $\Omega_{k}$ with k=1,2,...,10 (see Table 9).

#### 4.3.6. $p_{i}$ Based on Distance

Chen et al. [28] proposed a graph entropy metric while using an information functional (see Equation (9)) based on distance, which they claimed “is one of the most important graph invariants”. Their metric considers the number of vertices with distance *l* to a given vertex, with 1≤l≤D(G) where D(G) is the diameter of graph *G*. Thus $p_{i}=n_{l}\left(i\right)/\sum_{j=1}^{N}n_{l}\left(j\right)$.

Singh et al. [35] introduced an entropy metric that measures the influence of links in the network by determining the average path length with and without the presence of an edge (i,j). For this, $p_{i}=p_{ij}=|APL-APL_{ij}|/\sum_{i\neq j}\left|APL-APL_{ij}\right|$, i.e., it is proportional to the absolute difference in average path length between the original graph and after the removal of edge (i,j). In addition, the authors extended the metric in order to determine the influence of a node *i* on the network while using $p_{i}=0.5\sum_{j}p_{ij}$.

Finally, Stella et al. [47] proposed a metric, called distance entropy, which quantifies node centrality through the distribution of path lengths. In this case, $p_{i}=p_{l}^{i}=n_{l}/\left(N-1\right)$, where $n_{l}$ is the number of nodes at a distance *l* from node *i*. The summation in Shannon’s entropy formulation (see Equation (10)) is conducted between l=1 and the difference between the maximum and minimum distance between node *i* and any other node *j* in the network. Unlike most of the metrics presented in this review article, the more central a node, the lower its entropy. This is because distance entropy measures the regularity of paths lengths between a node and its neighbors.

A summary of these metrics can be found in Table 10.

#### 4.3.7. $p_{i}$ Based on Eigenvector

Jimenez et al. [40] quantified the entropy and connectivity of porous media with a particular flow direction. They used Shannon’s entropy formulation, where the probability distribution is based on eigenvector centrality. In this case, $p_{i}=p\left(x_{i}\right)$, i.e., the probability of a node in the pore network with eigenvector centrality value $x_{i}$. The authors claimed that this metric could quantify the impact of water saturation, given that they observed that entropy increases as saturation decreases.

Zarghami et al. [53] developed a vulnerability index in order to evaluate water distribution networks. The index is based on eigenvector as well as closeness and betweenness centrality (see Section 4.3.2 and Section 4.3.5). The eigenvector portion makes used of information functionals (see Equation (9)), and, thus, $p_{i}=x_{i}/\sum_{j}x_{j}$.

A summary of these metrics can be found in Table 11.

#### 4.3.8. Other $p_{i}$ Definitions

A number of authors have proposed entropy metrics whose probability distributions do not correspond to any of the groups discussed previously. Table 12 summarizes these metrics. Hussain et al. [15] based the probability distribution on Bayes posterior probability, although it is unclear how they calculate it. Sun et al. [20] used an information functional that is based on the topological potential, which is a function of the strength of a node, the shortest paths between node pairs, and an optimized impact factor. Weber et al. [36] localized fault producing process steps in integrated circuit manufacturing lines. In this case, $p_{i}$ is defined as the probability of a fault occurring in process step *i*. Wang et al. [38], as explained in Section 4.3.5, developed a combined metric. The first part is calculated while using the sum of the closeness centrality and the clustering coefficient. Xu et al. [51] presented the origin-destination entropy with flow to rank road intersections. Their method requires a tripartite graph with three distinctive sets of nodes (origin-destination pairs, paths, and intersections) and defines $p_{i}=p_{s}\left(i\right)$ as the probability that the flow on node *i* is from the origin-destination pair *s*. Zareie et al. [52] used the diversity strength ranking, which is a function of the improved k-shell (IKS) of node *i* and the sum of the IKS of the neighbors of *i*. Thus, $p_{i}=IKS\left(i\right)/\sum_{j\in \Gamma (i)}IKS\left(j\right)$. Wen et al. [58] used the fraction of nodes that are contained in a box of size *l* around node *i* giving $p_{i}=n\left(i,l\right)/N$. Finally, some authors used entropy to study complex networks that represent proteins. Zhao et al. [55] predicted essential proteins from protein interaction networks while using Shannon’s entropy. Bashiri et al. [61] used node annotations regarding protein functions, diseases, and drugs that target them as the basis for identifying important proteins in protein interaction networks.

### 4.4. Metric Applicability

Complex networks can be either directed, where $\left(v_{i},v_{j}\right)\neq \left(v_{j},v_{i}\right)$ for $\left(v_{i},v_{j}\right),\left(v_{j},v_{i}\right)\in E$, or undirected where $\left(v_{i},v_{j}\right)\equiv \left(v_{j},v_{i}\right)$, as explained in Section 2. Furthermore, graphs can be binary or unweighted if described by an adjacency matrix A of elements $a_{ij}\in \left\{0,1\right\}$; or, weighted if described by a weight matrix W of elements $w_{ij}>0$. The records included in this literature review propose entropy based metrics mostly for unweighted (65% of records) and undirected (69% of records) graphs, as shown in Table 13. Only a small portion of records specify other network characteristics, such as requiring the graph to be acyclic (two records), aperiodic (one record), connected (two records), or strongly connected (one record), as well as prohibiting the existence of self-loops (three records). This result highlights the necessity to extend existing network entropy metrics to weighted, directed graphs, and/or to develop appropriate methods for them.

## 5. Discussion

This narrative review has identified a number of ways to classify information entropy metrics for complex networks. In Section 4.1, entropy metrics were grouped, depending on whether they constituted a graph H(G) or a node H(i) property. Graph entropy metrics provide a single value in order to characterize the full graph. On the other hand, node entropy metrics share characteristics with traditional centrality metrics in graph theory, facilitating the generation of rankings that are typically used to identify important nodes. However, it was shown in Section 4.2 that graph entropy metrics have been indirectly used in order to also produce node rankings. This is generally accomplished by calculating the difference between the entropy of the full graph and the entropy of the graph when a node and its adjacent edges are removed. The procedure is followed for all nodes in the graph in order to identify the node that generates the biggest change in entropy. Consequently, a ranking is indirectly produced.

Section 4.3 explores a different way to classify information entropy metrics for complex networks, where the focus was on the underlying probability distributions. This section demonstrated that most of the research efforts have been allocated to degree (or strength) based entropy metrics. The use of other probability distributions trails greatly behind with betweenness centrality, paths, and walks in second place. However, it must be noted that betweenness and paths suffer from computational complexity for moderate and big graphs, making them less attractive. While algorithms for calculating betweenness centrality required originally $O(|V{|}^{3})$ time and $O(|V{|}^{2})$ memory space, Brandes revolutionary work [65] reduced these requirements to O(|V||E|) time and O(|V|+|E|) memory space for unweighted graphs and to $O(|V||E|+|V{|}^{2}\log|V|)$ time and O(|V|+|E|) memory space for weighted ones. Yet, path-based entropy centrality metrics still require the search of all paths in *G* (or, at least, the search for paths with a probability above a certain user defined threshold) hindering adoption [14,17,29,46,54]. Thus, the best alternative to paths up to date, at least in terms of computational complexity, is to use walks given that the entropy can be calculated exactly while using matrix operations [29]. Other probability distributions, such as those that are based on closeness centrality, distances in graphs, eigenvector centrality, k-shells, and/or clustering coefficient, among others, have been seldom explored leaving space for further development.

Finally, information entropy metrics for complex networks can be classified based on the types of graphs to which they are applicable, as in Section 4.4. This narrative literature review showed that research efforts have mostly focused on undirected, unweighted networks. The biggest downside (as well as the biggest opportunity for further research) is that many interesting complex interactions are represented as either directed and/or weighted graphs. Thus, it is evident that further development of existing metrics to extend their applicability is necessary.

In conclusion, it must be recognized that, although the development of information entropy metrics for complex networks has been ongoing for 15 years, the existing body of research is limited. Thus, it can be fairly argued that the field is in its infancy. This feeling is shared by other authors. In fact, Ni et al. [60] stated, as recently as 2020, that, in particular, "research on entropy based centrality is still in a nascent stage". It is also opportunely to add that research on the underlying assumptions of entropy metrics and the context in which they are applicable also requires more thorough studies.

## Figures and Tables

**Table 1:** Included records, by year.

Refs.	Authors	Year	Record Type	Journal/Booktitle
[14]	Tutzauer, Frank	2007	article	Social networks
[15]	Hussain, DM Akbar and Ortiz-Arroyo, Daniel	2008	proceeding	European Conference on Intelligence and Security Informatics
[16]	Ortiz-Arroyo, Daniel and Hussain, DM Akbar	2008	proceeding	European Conference on Intelligence and Security Informatics
[17]	Tutzauer, Frank and Elbirt, Benjamin	2009	article	Communication Monographs
[18]	Dehmer, Matthias and Mowshowitz, Abbe	2011	article	Information Sciences
[19]	Delvenne, Jean-Charles and Libert, Anne-Sophie	2011	article	Physical Review E
[20]	Sun, Rui and Mu, A-li and Li, Lin and Zhong, Mi	2012	proceeding	Fourth International Conference on Machine Vision (ICMV 2011): Machine Vision, Image Processing, and Pattern Analysis
[21]	Serin, Ekrem and Balcisoy, Selim	2012	proceeding	2012 IEEE/ACM International Conference on Advances in Social Networks Analysis and Mining
[22]	Dehmer, Matthias and Sivakumar, Lavanya	2012	article	PloS one
[23]	Fewell, Jennifer H and Armbruster, Dieter and Ingraham, John and Petersen, Alexander and Waters, James S	2012	article	PloS one
[24]	Chellappan, Vanniarajan and Sivalingam, Krishna M	2013	proceeding	2013 19th IEEE Workshop on Local & Metropolitan Area Networks (LANMAN)
[25]	V. Chellappan and K. M. Sivalingam and K. Krithivasan	2014	proceeding	2014 IEEE 20th International Workshop on Local Metropolitan Area Networks (LANMAN)
[26]	Estrada, Ernesto and José, A and Hatano, Naomichi	2014	article	Linear Algebra and its Applications
[27]	Benzi, Michele	2014	article	Linear Algebra and its Applications
[28]	Chen, Zengqiang and Dehmer, Matthias and Shi, Yongtang	2014	article	Entropy
[29]	Nikolaev, Alexander G and Razib, Raihan and Kucheriya, Ashwin	2015	article	Social Networks
[30]	Caravelli, Francesco	2015	article	Chaos, Solitons & Fractals
[31]	Lu, Guoxiang and Li, Bingqing and Wang, Lijia	2015	article	Entropy
[32]	Nie, Tingyuan and Guo, Zheng and Zhao, Kun and Lu, Zhe-Ming	2016	article	Physica A: Statistical Mechanics and its Applications
[33]	Gialampoukidis, Ilias and Kalpakis, George and Tsikrika, Theodora and Vrochidis, Stefanos and Kompatsiaris, Ioannis	2016	proceeding	2016 European Intelligence and Security Informatics Conference (EISIC)
[34]	Chellappan, Vanniyarajan and Sivalingam, Krishna M and Krithivasan, Kamala	2016	article	Computer Networks
[35]	Singh, Priti and Chakraborty, Abhishek and Manoj, BS	2017	article	Physica A: Statistical Mechanics and its Applications
[36]	Weber, Charles M and Hasenauer, Rainer P and Mayande, Nitin V	2017	proceeding	2017 Portland international conference on management of engineering and technology (PICMET)
[37]	Bekiros, Stelios and Nguyen, Duc Khuong and Junior, Leonidas Sandoval and Uddin, Gazi Salah	2017	article	European Journal of Operational Research
[38]	Wang, Qin and Zeng, Guangping and Tu, Xuyan	2017	article	Entropy
[39]	Ai, Xinbo	2017	article	Entropy
[40]	Jimenez-Martinez, Joaquin and Negre, Christian FA	2017	article	Physical Review E
[41]	Cai, Meng and Cui, Ying and Stanley, H Eugene	2017	article	Scientific reports
[42]	Wiedermann, Marc and Donges, Jonathan F and Kurths, Jürgen and Donner, Reik V	2017	article	Physical Review E
[43]	Zareie, Ahmad and Sheikhahmadi, Amir and Fatemi, Adel	2017	article	Chaos, Solitons & Fractals
[44]	Qiao, Tong and Shan, Wei and Zhou, Chang	2017	article	Entropy
[45]	Tulu, Muluneh Mekonnen and Hou, Ronghui and Younas, Talha	2018	article	IEEE Access
[46]	Oggier, Frédérique and Phetsouvanh, Silivanxay and Datta, Anwitaman	2018	proceeding	2018 International Symposium on Information Theory and Its Applications (ISITA)
[47]	Stella, Massimo and De Domenico, Manlio	2018	article	Entropy
[48]	Qiao, Tong and Shan, Wei and Yu, Ganjun and Liu, Chen	2018	article	Entropy
[49]	Barucca, Paolo and Caldarelli, Guido and Squartini, Tiziano	2018	article	Journal of Statistical Physics
[50]	Zhang, Zundong and Ma, Weixin and Zhang, Zhaoran and Xiong, Changzhe	2018	proceeding	2018 Chinese Control And Decision Conference (CCDC)
[51]	M. Xu and J. Wu and M. Liu and Y. Xiao and H. Wang and D. Hu	2019	article	IEEE Transactions on Intelligent Transportation Systems
[52]	Ahmad Zareie and Amir Sheikhahmadi and Mahdi Jalili	2019	article	Future Generation Computer Systems
[53]	Zarghami, Seyed Ashkan and Gunawan, Indra and Schultmann, Frank	2019	article	Built Environment Project and Asset Management
[54]	Oggier, Frédérique and Phetsouvanh, Silivanxay and Datta, Anwitaman	2019	article	PeerJ Computer Science
[55]	J. Zhao and X. Lei	2019	article	IEEE Access
[56]	Wang, Lixiang and Dai, Wei and Luo, Guixiu and Zhao, Yu	2019	article	Entropy
[57]	Li, Yichuan and Cai, Weihong and Li, Yao and Du, Xin	2020	article	Entropy
[58]	Tao Wen and Yong Deng	2020	article	Information Sciences
[59]	Guo, Chungu and Yang, Liangwei and Chen, Xiao and Chen, Duanbing and Gao, Hui and Ma, Jing	2020	article	Entropy
[60]	Ni, Chengzhang and Yang, Jun and Kong, Demei	2020	article	Physica A: Statistical Mechanics and its Applications
[61]	Hamid Bashiri and Hossein Rahmani and Vahid Bashiri and Dezső Módos and Andreas Bender	2020	article	Computers in Biology and Medicine
[62]	Min Wang and Wanchun Li and Yuning Guo and Xiaoyan Peng and Yingxiang Li	2020	article	Physica A: Statistical Mechanics and its Applications
[63]	Chandni Saxena and M.N. Doja and Tanvir Ahmad	2020	article	Physica A: Statistical Mechanics and its Applications

**Table 2:** Summary of entropy metrics based on degree.

Refs.	Entropy Formulation	Notes
[16]	$H\left(G\right)=-\sum_{i=1}^{N}\frac{k_{i}}{2N}\log_{2}\frac{k_{i}}{2N}$	Note that, $\frac{k_{i}}{2N}=\frac{k_{i}}{\sum_{j=1}^{N}k_{j}}$. Thus, Ortiz’ formulation is equivalent to that of [39,41,56].
[21]	$H\left(i\right)=-\sum_{i=1}^{N}\frac{k_{i}^{\mathrm{norm}}}{\sum_{j=1}^{N}k_{j}^{\mathrm{norm}}}\log\frac{k_{i}^{\mathrm{norm}}}{\sum_{j=1}^{N}k_{j}^{\mathrm{norm}}}$	$k_{i}^{\mathrm{norm}}=\frac{k_{i}-k_{\min}}{k_{\max}-k_{\min}}$
[31]	$H\left(G\right)=-\sum_{i=1}^{N}\frac{k_{i}^{q}}{\sum_{j=1}^{N}k_{j}^{q}}\log\frac{k_{i}^{q}}{\sum_{j=1}^{N}k_{j}^{q}}$	$k_{i}^{q}$ is the *q* degree power of node *i*.
[39,41,56]	$H\left(G\right)=-\sum_{i=1}^{N}\frac{k_{i}}{\sum_{j=1}^{N}k_{j}}\log\frac{k_{i}}{\sum_{j=1}^{N}k_{j}}$	Identical to [31] with q=1. Note that [41] uses the natural logarithm.
[41]	$H\left(G\right)=-\sum_{k=0}^{N-1}p\left(k\right)\ln p\left(k\right)$	p(k) is the distribution function of the degree.
[41]	$H\left(G\right)=-\sum_{i=1}^{N}p_{i}\ln p_{i}$	$p_{i}=\frac{\left(k_{i}+1\right)\left[1-p\left(k_{i}\right)+\Delta \right]}{\sum_{j=1}^{N}\left(k_{j}+1\right)\left[1-p\left(k_{j}\right)+\Delta \right]}$ where $p(k_{i})$ is the distribution probability of node degree $k_{i}$ and $\Delta ∼O(1/N^{2})$.
[41]	$H\left(G\right)=-\sum_{l=1}^{N}p_{l}\ln p_{l}$	$p_{l}=\frac{\sum_{\left(i,j\in S(l)\right)}{(}^{l}W_{i,j}{-}^{l}W_{i,j}^{*})+k_{l}+\Delta }{\sum_{n=1}^{N}\left[\sum_{\left(i,j\in S(n)\right)}{(}^{n}W_{i,j}{-}^{n}W_{i,j}^{*}+\Delta )+k_{n}\right]}$ where S(l)={(i,j):1≤i≤N;1≤j≤N;i≠j≠l}, $\Delta ∼O(1/N^{2})$, *W* is the maximum flow matrix and ${}^{l}W$ is the matrix when row *l* and column *l* are removed from *W*.
[42]	$H\left(i\right)=-\sum_{j=1}^{N}\frac{a_{ij}}{k_{i}}\log\frac{a_{ij}}{k_{i}}$	
[45]	$H\left(i\right)=\left[-\sum \rho_{i}^{\mathrm{in}}\log\rho_{i}^{\mathrm{in}}\right]+\left[-\sum \rho_{i,h_{1}}^{\mathrm{ext}}\log\rho_{i,h_{1}}^{\mathrm{ext}}\right]$	where $\rho_{i}^{\mathrm{in}}=\frac{\sum_{j}a_{ij}}{k_{i}}$ for i,j∈h, and $\rho_{i,h_{1}}^{\mathrm{ext}}=\frac{\sum_{j}a_{ij}}{k_{i}}$ for i∈h & $j\in h_{1}$.
[49]	$H\left(G\right)=-\frac{1}{2}\sum_{i}\sum_{j\neq i}\left[p_{ij}\ln p_{ij}+\left(1-p_{ij}\right)\ln\left(1-p_{ij}\right)\right]$	$p_{ij}=\frac{x_{i}x_{j}}{1+x_{i}x_{j}}$ is the Configuration Model representation.

**Table 3:** Summary of entropy metrics based on strength.

Refs.	Entropy Formulation	Notes
[38]	$H\left(i\right)=-\sum_{i=1}^{m_{j}}\frac{s_{i}}{\sum_{j=1}^{m_{j}}s_{j}}\ln\frac{s_{i}}{\sum_{j=1}^{m_{j}}s_{j}}$	$m_{j}$ is the number of projects in community *j*.
[56,60]	$H\left(i\right)=-\sum_{j\in \Gamma (i)}\frac{w_{ij}}{s_{i}}\log\frac{w_{ij}}{s_{i}}$	Note that in this case the summation over j∈Γ(i) produces the same result as conducted over j∈V.

**Table 4:** Summary of entropy metrics based on degree or strength of neighbors.

Refs.	Entropy Formulation	Notes
[32]	$H\left(i\right)=-k_{i}\sum_{j\in \Gamma (i)}\log k_{j}$	Note that this is not strictly based on Shannon’s entropy.
[43,59]	$H_{1}\left(i\right)=-\sum_{j\in \Gamma (i)}\frac{k_{j}}{k_{i}^{1}}\log\frac{k_{j}}{k_{i}^{1}}$	$k_{i}^{1}=\sum_{j\in \Gamma (i)}k_{j}$ is the total degree of the neighbors of node *i*.
[43]	$H_{2}\left(i\right)=-\sum_{j\in \Gamma (i)}\frac{k_{j}^{1}}{k_{i}^{2}}\log\frac{k_{j}^{1}}{k_{i}^{2}}$	$k_{i}^{2}=\sum_{j\in \Gamma (i)}k_{j}^{1}$ is the total degree of the neighbors of node *i*’s neighbors.
[57]	$H^{x}\left(i\right)=-\sum_{i=1}^{N}\frac{k_{i}^{x}}{\sum_{j\in \Gamma (i)}k_{j}^{x}}\log\frac{k_{i}^{x}}{\sum_{j\in \Gamma (i)}k_{j}^{x}}$	*x* represents either the “in” or “out” component of the degree.
[57]	$H^{x}\left(i\right)=-\sum_{i=1}^{N}\frac{s_{i}^{x}}{\sum_{j\in \Gamma (i)}s_{j}^{x}}\log\frac{s_{i}^{x}}{\sum_{j\in \Gamma (i)}s_{j}^{x}}$	*x* represents either the “in” or “out” component of the strength.
[60]	$H\left(i\right)=-\sum_{j\in \Gamma (i)}\frac{k_{j}^{\beta }}{\sum_{l\in \Gamma (i)}k_{l}^{\beta }}\log\frac{k_{j}^{\beta }}{\sum_{l\in \Gamma (i)}k_{l}^{\beta }}$	“confidence influence entropy”
[62]	$H\left(i\right)=-\sum_{j\in \Gamma (i)}\frac{k_{j}}{\sum_{l=1}^{N}k_{l}}\ln\frac{k_{j}}{\sum_{l=1}^{N}k_{l}}$	
[63]	$H\left(i\right)=-\sum_{j\in \Gamma (i)}\frac{1}{k_{i}\left(k_{j}-1\right)}\log\frac{1}{k_{i}\left(k_{j}-1\right)}$	

**Table 5:** Summary of entropy metrics that are based on degree or strength in subgraphs.

Refs.	Entropy Formulation	Notes
[44,48]	$H\left(i\right)=-\sum_{i\in G_{i}}\frac{k_{i}^{G_{i}}}{\sum_{j\in G_{i}}k_{j}^{G_{i}}}\log\frac{k_{i}^{G_{i}}}{\sum_{j\in G_{i}}k_{j}^{G_{i}}}$	$G_{i}$ is the subgraph that has node *i* as central node.
[48]	$H\left(i\right)=-\sum_{j\in G_{i}}\frac{w_{ij}^{G_{i}}}{\sum_{l\in G_{i}}w_{il}^{G_{i}}}\log\frac{w_{ij}^{G_{i}}}{\sum_{l\in G_{i}}w_{il}^{G_{i}}}$	$G_{i}$ is the subgraph that has node *i* as central node.

**Table 6:** Summary of entropy metrics based on betweenness centrality.

Refs.	Entropy Formulation	Notes
[21]	$H\left(i\right)=-\sum_{i=1}^{N}\frac{\eta_{i}^{\mathrm{norm}}}{\sum_{j=1}^{N}\eta_{j}^{\mathrm{norm}}}\log\frac{\eta_{i}^{\mathrm{norm}}}{\sum_{j=1}^{N}\eta_{j}^{\mathrm{norm}}}$	$\eta_{i}^{\mathrm{norm}}=\frac{\eta_{i}-\eta_{\min}}{\eta_{\max}-\eta_{\min}}$
[24,25,34]	$H\left(G\right)=-\sum_{(u,v)\in E}p\left(u,v\right)\log p\left(u,v\right)$	$p\left(u,v\right)=\frac{\eta_{•,•}\left(u,v\right)}{\sum_{(x,y)\in E}\eta_{•,•}\left(x,y\right)}$ where $\eta_{•,•}\left(u,v\right)$ is the shortest path betweenness centrality of a link (u,v) for every pair of source-sink nodes.
[33]	$H\left(i\right)=-\eta_{i}\sum_{j\in \Gamma (i)}\log\eta_{j}$	Not strictly following Shannon’s formulation.
[39,53]	$H\left(G\right)=-\sum_{i=1}^{N}\frac{\eta_{i}}{\sum_{j=1}^{N}\eta_{j}}\log\frac{\eta_{i}}{\sum_{j=1}^{N}\eta_{j}}$	
[50]	$H\left(G\right)=-\sum_{(u,v)\in E,\forall i,\forall j}\frac{\eta_{i,j}\left(u,v\right)}{\eta_{i,j}}\ln\frac{\eta_{i,j}\left(u,v\right)}{\eta_{i,j}}$	While similar to the formulation in [24,25,34], the chosen logarithm base is different.

**Table 7:** Summary of entropy metrics based on paths.

Refs.	Entropy Formulation	Notes
[14,17]	$H\left(i\right)=-\sum_{j=1}^{N}p_{ij}\log_{2}p_{ij}$	$p_{ij}=\sum_{k=1}^{K(i,j)}\sigma_{k}\left(j\right)\prod_{t=0}^{n(k)-1}\tau_{k}\left(v_{t}\right)$ where $p_{ij}$ is the probability of a path starting in node *i* and ending on node *j* which is a function of the transfer $\tau_{k}\left(v_{t}\right)$ and the stopping $\sigma_{k}\left(j\right)$ probabilities.
[16]	$H\left(G\right)=-\sum_{i=1}^{N}\gamma \left(v_{i}\right)\log_{2}\gamma \left(v_{i}\right)$	$\gamma \left(v_{i}\right)=\frac{\left|\mathrm{paths}(v_{i})\right|}{\left|\mathrm{paths}(v_{1},v_{2},...,v_{N})\right|}$ is the fraction of paths in graph *G* that start on node $v_{i}$.
[46]	$H\left(i\right)=-\sum_{j=1}^{N}p_{ij}\log_{2}p_{ij}$	$p_{ij}=\sum_{P\in P_{s,j}}\prod_{v\in P}\tau_{P_{v}}\left(v\right)\frac{f(v^{\prime },v)}{\left|S(P_{v})\right|}$ where *P* is a path in the set of paths between *s* and *j*, $P_{s,j}$. $\tau_{P_{v}}\left(v\right)$ is the split and transfer probability, $f(v^{\prime },v)$ is the flow incoming to node *v* and $\left|S(P_{v})\right|$ is the number of edges to which the flow can be split into.
[54]	$H\left(i\right)=-\sum_{v\in V}q_{uv}\log_{2}q_{uv}$	$q_{uv}=q\left(x\right)w_{x}\left(u,v\right)$ where q(x) is the probability of choosing an outgoing edge and $\sum_{x\in E_{u}}q\left(x\right)=1$. $w_{x}\left(u,v\right)$ is a weight associated with the edge (u,v) such that $\sum_{(u,v)\in x}w_{x}\left(u,v\right)=f_{u}$, i.e., the flow that reached node *u*.

**Table 8:** Summary of entropy metrics based on walks.

Refs.	Entropy Formulation	Notes
[19]	$H\left(i,t\right)=\lambda^{-t}u_{i}v_{j}\log\left(\lambda^{-t}u_{i}v_{j}\right)$	*t* is the path length, λ is the dominant eigenvalue of A, *u* is the left eigenvector and *v* is the right one.
[23]	N/A	Formulation is not provided by the authors.
[26,27]	$H\left(G\right)=-\sum_{i}\frac{{\left(\exp\beta A\right)}_{ii}}{Z}\ln\frac{{\left(\exp\beta A\right)}_{ii}}{Z}$	A is the adjacency matrix, $\beta ={\left(k_{B}T\right)}^{-1}$ is the inverse temperature and Z=Tr(expβA) is the partition function for the graph.
[29]	$H\left(i,t\right)=-\sum_{j=1}^{N}\left(p_{ij}^{\left(t\right)}+p_{ij^{\prime }}^{\left(t\right)}\right)\log\left(p_{ij}^{\left(t\right)}+p_{ij^{\prime }}^{\left(t\right)}\right)$	$p_{ij}$ are the elements of the Markov chain transition probability and *t* is the number of transitions.
[30]	$H\left(i\right)=-\frac{1}{N}\sum_{j=1}^{N}M_{ij}^{*}\log M_{ij}^{*}$	$M_{ij}^{*}=\lim_{k\to \infty }\sum_{j_{1},...,j_{k}=1}^{N}M_{ij_{1}}...M_{j_{k}j}$ with $M_{ij}$, the Markov operator.

**Table 9:** Summary of entropy metrics based on closeness centrality.

Refs.	Entropy Formulation	Notes
[21]	$H\left(i\right)=-\sum_{i=1}^{N}\frac{C_{i}^{norm}}{\sum_{j=1}^{N}C_{j}^{norm}}\log\frac{C_{i}^{norm}}{\sum_{j=1}^{N}C_{j}^{norm}}$	$C_{i}^{norm}=\frac{C_{i}-C_{min}}{C_{max}-C_{min}}$
[22,53]	$H\left(G\right)=-\sum_{i=1}^{N}\frac{C_{i}}{\sum_{j=1}^{N}C_{j}}\log\frac{C_{i}}{\sum_{j=1}^{N}C_{j}}$	
[38]	$H\left(i\right)=-\sum_{k=1}^{10}p_{k}\ln p_{k}$	$p_{k}=\sum_{j}p\left(\mu_{j}\right)$ where $\mu_{j}\in \Omega_{k}$ and $\mu_{j}=\beta_{1}C_{j}+\beta_{2}C_{C}\left(j\right)$. The range of μ is [0,1] and is divided into ten intervals $\Omega_{k}$ with k=1,2,...,10.

**Table 10:** Summary of entropy metrics based on distance.

Refs.	Entropy Formulation	Notes
[28]	$H\left(G\right)=-\sum_{i=1}^{N}\frac{n_{l}\left(i\right)}{\sum_{j=1}^{N}n_{l}\left(j\right)}\log\frac{n_{l}\left(i\right)}{\sum_{j=1}^{N}n_{l}\left(j\right)}$	$n_{l}$ is the number of vertices with distance *l* to a given vertex.
[35]	$H\left(G\right)=-\sum_{i\neq j}\frac{|APL-APL_{ij}|}{\sum_{i\neq j}\left|APL-APL_{ij}\right|}\log\frac{|APL-APL_{ij}|}{\sum_{i\neq j}\left|APL-APL_{ij}\right|}$	$APL=2\sum_{i\neq j}\frac{d_{ij}}{N(N-1)}$ where $d_{ij}$ is the distance of the path between *i* and *j*.
[35]	$H\left(G\right)=-\sum_{i\neq j}p_{i}\log p_{i}$	$p_{i}=\frac{1}{2}\sum_{j}\frac{|APL-APL_{ij}|}{\sum_{i\neq j}\left|APL-APL_{ij}\right|}$
[47]	$H\left(i\right)=\frac{-1}{\log(M_{i}-m_{i})}\sum_{l=1}^{M_{i}-m_{i}}\frac{n_{l}}{N-1}\log\frac{n_{l}}{N-1}$	$M_{i}=\max_{j}d_{ij}$ and $m_{i}=\min_{j}d_{ij}$.

**Table 11:** Summary of entropy metrics based on eigenvector centrality.

Refs.	Entropy Formulation	Notes
[40]	$H\left(G\right)=-\sum_{i=1}^{N}p\left(x_{i}\right)\log p\left(x_{i}\right)$	$p(x_{i})$ is the probability of a node with eigenvector centrality value $x_{i}$.
[53]	$H\left(G\right)=-\sum_{i=1}^{N}\frac{x_{i}}{\sum_{j}x_{j}}\log\frac{x_{i}}{\sum_{j}x_{j}}$	

**Table 12:** Summary of entropy metrics based on other probability distributions

Refs.	Entropy Formulation	Notes
[15]	$H\left(G\right)=-\sum_{i=1}^{N}p_{i}\log p_{i}$	Based on Bayes posterior probability but it is unclear how $p_{i}$ is obtained.
[20]	$H\left(G,\sigma \right)=-\sum_{i=1}^{N}\frac{\varphi_{i}}{\sum_{j=1}^{N}\varphi_{j}}\log\frac{\varphi_{i}}{\sum_{j=1}^{N}\varphi_{j}}$	$\varphi_{i}=\sum_{j=1}^{N}s_{j}\exp{\left(\frac{-d_{ij}}{\sigma }\right)}^{2}$ is the topological potential which is a function of the strength $s_{j}$, the shortest path between all pairs of nodes $d_{ij}$ and an optimized impact factor σ.
[36]	$H\left(i\right)=-\sum_{i=1}^{N}P\left(X_{i}\right)\log P\left(X_{i}\right)$	*i* is a process step, $X_{i}$ is the event that the fault is produced in step *i* and $P(X_{i})$ is the probability of this fault occurring in $X_{i}$.
[38]	$H\left(i\right)=-\sum_{k=1}^{10}p_{k}\ln p_{k}$	$p_{k}=\sum_{j}p\left(\mu_{j}\right)$ where $\mu_{j}\in \Omega_{k}$ and $\mu_{j}=\beta_{1}C_{j}+\beta_{2}C_{C}\left(j\right)$. The range of μ is [0,1] and is divided into ten intervals $\Omega_{k}$ with k=1,2,...,10.
[51]	$H\left(i\right)=-\sum_{s=1}^{\left|S\right|}p_{s}\left(i\right)\log p_{s}\left(i\right)$	|S| is the total number of origin-destination pairs, $p_{s}\left(i\right)$ is the probability that the flow on node *i* is from origin-destination pair *s*.
[52]	$H\left(i\right)=-\sum_{j\in \Gamma (i)}\frac{IKS(j)}{\sum_{l\in \Gamma (i)}IKS\left(l\right)}\log\frac{IKS(j)}{\sum_{l\in \Gamma (i)}IKS\left(l\right)}$	IKS is the improved k-shell index.
[55]	$H\left(i\right)=-\sum_{j=1}^{nc}{\mathrm{ComInf}}_{i}\left(j\right)p\left(c_{j}\right)\log p\left(c_{j}\right)$	${\mathrm{ComInf}}_{i}\left(j\right)$ is an nc dimensional vector representing the protein associated complex information (${\mathrm{ComInf}}_{i}\left(j\right)=1(if)v_{i}\in c_{j},\mathrm{else}0$. The probability of a protein complex $p\left(c_{j}\right)=|c_{j}\left|/|C\right|$ where $|c_{j}|$ is the number of proteins contained in the protein complex $c_{j}$ and |C| is the number of proteins in the standard protein complex *C*.
[55]	$H\left(i\right)=-\sum_{k=1}^{ns}{\mathrm{SubInf}}_{i}\left(k\right)p\left(s_{k}\right)\log p\left(s_{k}\right)$	${\mathrm{SubInf}}_{i}\left(k\right)$ is an ns dimensional vector representing the protein associated subcellular localization information (${\mathrm{SubInf}}_{i}\left(k\right)=1(if)v_{i}\in s_{k},\mathrm{else}0$. The probability of a protein complex $p\left(s_{k}\right)=|s_{k}\left|/|C\right|$ where $|s_{k}|$ is the number of proteins contained in the protein complex $s_{k}$ and |C| is the number of proteins in the standard protein complex *C*.
[58]	$I\left(i,l\right)=-\frac{n(i,l)}{N}\ln\frac{n(i,l)}{N}$	Note that this is the information of a box of size *l* around node *i*, not the entropy.
[61]	$H\left(i\right)=-\sum_{j=1}^{N}p_{i}\log p_{i}$	$p_{i}=\frac{{\mathrm{AnnContx}}_{i}^{j}}{\sum_{l=1}^{\left|\mathrm{AnnContx}\right|}{\mathrm{AnnContx}}_{i}^{l}}$ where *i* is a node or protein, *N* is the number of annotations in the annotation list and ${\mathrm{AnnContx}}_{i}^{j}=\sum_{l\in NB(i)}{\mathrm{AnnList}}_{l}^{j}$ where the value of ${\mathrm{AnnList}}_{l}^{j}$ is 1 or 0 depending on *i* being annotated by annotation $a_{j}$.

**Table 13:** Graph types to which the entropy metric is applicable.

Refs.	Undirected	Directed	Unweighted	Weighted	Other Requirements
[14]	X	X	X	X	
[15]	X		X		acyclic
[16]	X		X		acyclic
[17]	X	X	X	X	
[18]	X		X		
[19]		X	X		strongly connected, aperiodic
[20]	X			X	connected
[21]	X		X		
[22]	X		X		
[23]		X		X	
[24]		X	X		no self-loops
[25]		X		X	
[26]	X		X		
[27]	X		X		
[28]	X		X		no self-loops
[29]	X		X	X	
[30]	X			X	
[31]	X		X		
[32]	X		X		
[33]	X		X		
[34]	X			X	
[35]	X			X	
[36]		X	X		
[37]	X		X		
[38]	X		X		
[39]		X	X		
[40]	X			X	
[41]	X		X		
[42]	X		X		no self-loops
[43]	X		X		
[44]	X		X		
[45]	X		X		
[46]		X	X		
[47]	X		X		connected
[48]		X		X	
[49]	X	X	X	X	
[50]		X		X	
[51]		X		X	
[52]	X		X		
[53]	X	X	X		
[54]		X		X	
[55]	X			X	
[56]		X		X	
[57]		X		X	
[58]					not specified
[59]	X		X		
[60]	X			X	
[61]	X		X		
[62]	X		X		
[63]	X		X		

## Abbreviations

The following abbreviations and symbols are used in this manuscript:

**Greek Letters**	
β	Tunable parameter called confidence strength used in [60]
Γ(i)	Neighbors of node *i*
$\eta_{i}$	Betweenness centrality of node *i*
λ	Largest eigenvalue of $A=a_{ij}$
σ(i,j)	Total number of geodesic paths between nodes *i* and *j*
σ(i,j|v)	Number of geodesic paths between nodes *i* and *j* that pass through *v*
**Roman Letters**	
$A=[a_{ij}]$	Adjacency matrix of elements $a_{ij}$ used to describe graph *G*
$C_{i}$	Closeness centrality of node *i*
d(i,j)	Distance between nodes *i* and *j*
*E*	Set of edges in graph *G*
*f*	Abstract information function of *G*
*G*	Graph
$G_{i}$	Subgraph where node *i* is the central node
*H*	Information or Shannon’s entropy
IKS(i)	Improved k-shells of node *i*
$k_{i}$	Degree of node *i*
$k_{i}^{\mathrm{in}}$	In-degree of node *i*
$k_{i}^{\mathrm{out}}$	Out-degree of node *i*
$k_{\max}$	Maximum degree in graph *G*
$k_{\min}$	Minimum degree in graph *G*
$k_{i}^{q}$	*q* degree power of node *i*
$k_{i}^{1}$	Sum of the degree of the neighbors of *i*, i.e., $k_{i}^{1}=\sum_{j\in \Gamma (i)}k_{j}$
$k_{i}^{2}$	Sum of the degree of the neighbors of *i*’s neighbors, i.e., $k_{i}^{2}=\sum_{j\in \Gamma (i)}k_{j}^{1}$
$k_{j}^{G_{i}}$	Degree of node *j* in the subgraph that has node *i* as central node
*N*	Number of nodes in *G*, N=|V|
$p_{i}$	Probability of an outcome
$s_{i}$	Strength of node *i*
$s_{i}^{\mathrm{in}}$	In-strength of node *i*
$s_{i}^{\mathrm{out}}$	Out-strength of node *i*
*V*	Set of edges in graph *G*, V≠∅, $V=\{v_{0},v_{1},...,v_{N}\}$
$W=[w_{ij}]$	Weight matrix of elements $w_{ij}$ used to describe *G*
$x_{i}$	Eigenvector centrality of node *i*
